# Sellar Solitary Fibrous Tumor Mimicking Pituitary Adenoma: Diagnostic Pitfalls, Contemporary Pathological Classification, and Management Considerations

**DOI:** 10.3390/diagnostics16142161

**Published:** 2026-07-10

**Authors:** Ozan Baskurt, Mehmet Arda Inan, Kubilay Ukinc, Nurperi Gazioglu

**Affiliations:** 1 Department of Neurosurgery, Acibadem Maslak Hospital, Istanbul 34457, Turkey; 2 Department of Pathology, Acibadem University School of Medicine, Istanbul 34752, Turkey; drmardainan@gmail.com; 3 Department of Endocrinology and Metabolism, Istinye University, Istanbul 34396, Turkey; kubilay.ukinc@istinye.edu.tr; 4 Pituitary Center, Istanbul University-Cerrahpasa, Istanbul 34320, Turkey; nurperi.gazioglu@gmail.com

**Keywords:** solitary fibrous tumor, sellar tumor, STAT6 positive, differential diagnosis, pituitary adenoma

## Abstract

**Background/Objectives**: Sellar solitary fibrous tumors (SFTs) are exceptionally rare mesenchymal neoplasms that frequently mimic non-functioning pituitary adenomas (PAs) because of overlapping clinical manifestations and nonspecific radiological findings. Consequently, preoperative diagnosis remains challenging and definitive diagnosis relies on histopathological and immunohistochemical evaluation. **Methods**: We report a sellar SFT initially diagnosed as a PA and analyze the diagnostic features of previously reported cases to identify recurring diagnostic pitfalls. Clinical, endocrinological, radiological, intraoperative, histopathological, and immunohistochemical findings from a patient with a sellar SFT were retrospectively reviewed. A structured literature review of previously reported sellar SFTs was performed to compare presenting symptoms, endocrine abnormalities, imaging characteristics, pathological findings, and diagnostic features. **Results**: A 65-year-old man presented with headache, progressive visual impairment, fatigue, and anterior hypopituitarism. Magnetic resonance imaging demonstrated a heterogeneously enhancing sellar lesion with suprasellar extension and cavernous sinus involvement, leading to an initial diagnosis of non-functioning PA. Endoscopic transsphenoidal surgery revealed an unexpectedly hypervascular and firm tumor. Histopathological examination demonstrated a spindle-cell neoplasm with a hemangiopericytoma-like vascular pattern, six mitoses per 10 high-power fields, absence of necrosis, and diffuse nuclear STAT6 positivity, establishing the diagnosis of CNS WHO grade 2 solitary fibrous tumor according to the 2021 WHO classification. Review of the literature demonstrated that most sellar SFTs share similar clinical and radiological features with PAs and are diagnosed only after surgical resection. **Conclusions**: Sellar SFT should be considered in the differential diagnosis of atypical sellar masses despite the absence of characteristic imaging findings. Recognition of intraoperative features, together with appropriate immunohistochemical evaluation, particularly STAT6 staining, is essential for accurate diagnosis. Current evidence remains insufficient to define the optimal postoperative management of completely resected sellar SFTs, emphasizing the importance of individualized treatment decisions and long-term surveillance.

## 1. Introduction

Solitary fibrous tumor (SFT) is a rare fibroblastic mesenchymal neoplasm that can arise throughout the body, including the central nervous system (CNS) [1]. The identification of the NAB2–STAT6 gene fusion, resulting from a paracentric inversion on chromosome 12q13, established that lesions previously classified as SFT and hemangiopericytoma (HPC) represent a single biological entity [2]. This fusion leads to aberrant nuclear STAT6 expression, which serves as a highly sensitive and specific immunohistochemical surrogate marker for the diagnosis of SFT [2]. Consequently, the 2021 World Health Organization (WHO) Classification of Central Nervous System (CNS) Tumors unified these lesions under the designation SFT and introduced a grading system based on mitotic activity and tumor necrosis to better reflect biological behavior [2,3].

Intracranial SFTs are uncommon, accounting for less than 1% of primary CNS tumors, while involvement of the sellar or parasellar region is exceptionally rare [4,5]. Owing to their rarity, sellar SFTs are frequently overlooked in the differential diagnosis of sellar lesions. Patients typically present with nonspecific symptoms, including headache, visual impairment, and varying degrees of pituitary dysfunction, which closely resemble those observed in non-functioning (NF) pituitary adenomas (PAs) [6,7,8].

Radiological differentiation of sellar SFT from PA remains particularly challenging [9]. Conventional magnetic resonance imaging (MRI) usually demonstrates iso- to hypointense signal intensity on T1-weighted images, variable T2-weighted signal intensity, and heterogeneous contrast enhancement, findings that substantially overlap with those of PAs, meningiomas, and other sellar tumors [9,10,11,12]. Although advanced imaging techniques—including susceptibility-weighted imaging, dynamic contrast-enhanced MRI, diffusion-weighted imaging, and magnetic resonance spectroscopy—may provide additional information regarding tumor vascularity and tissue characteristics, no imaging feature has been shown to reliably distinguish sellar SFT from PA in routine clinical practice [13,14]. Consequently, definitive diagnosis continues to depend on histopathological examination and immunohistochemical confirmation.

Among immunohistochemical markers, nuclear STAT6 expression has emerged as the most sensitive and specific surrogate marker for the NAB2–STAT6 fusion and has become indispensable for the diagnosis of SFT [2]. The current WHO classification further emphasizes the importance of integrating histopathological findings with mitotic activity and necrosis for accurate grading and prognostic stratification [3]. Nevertheless, because of the rarity of sellar SFTs, evidence regarding their clinicoradiological presentation, optimal postoperative management, and long-term outcomes remains limited to isolated case reports and small case series.

In the present study, we report a sellar SFT that was initially diagnosed as an NF PA and was ultimately confirmed by histopathological and immunohistochemical evaluation. In addition to presenting the clinical course, radiological findings, surgical observations, and pathological features, we performed a structured review of previously reported sellar SFTs to identify recurring diagnostic patterns and to discuss current management considerations in the context of the contemporary WHO classification.

## 2. Materials and Methods

A 65-year-old man presented with a 6-month history of progressive headache, visual impairment, and generalized fatigue. Initial magnetic resonance imaging (MRI) performed at another institution demonstrated a sellar mass, and the patient was referred to our department for further evaluation.

Endocrinological assessment revealed anterior hypopituitarism, including markedly decreased testosterone, prolactin, and thyroid hormone levels, whereas morning cortisol and adrenocorticotropic hormone concentrations remained within the low-normal range (Table 1). The patient had previously received cabergoline for three months because of presumed hyperprolactinemia; this treatment was discontinued one week before admission.

**Figure 1:**
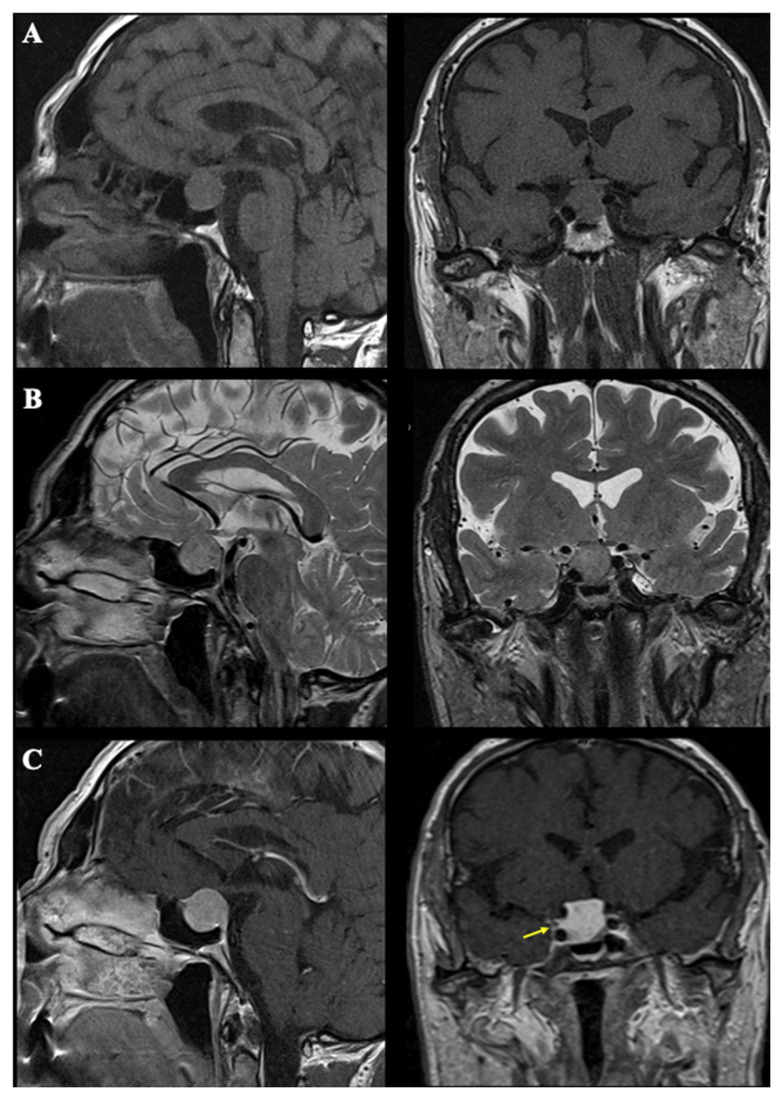
**Preoperative magnetic resonance imaging of the sellar solitary fibrous tumor.** (**A**) Sagittal (**left**) and coronal (**right**) T1-weighted images demonstrate an iso- to hypointense sellar mass with suprasellar extension. (**B**) Sagittal (**left**) and coronal (**right**) T2-weighted images show predominantly isointense signal intensity. (**C**) Contrast-enhanced sagittal (**left**) and coronal (**right**) T1-weighted images demonstrate heterogeneous enhancement with parasellar extension and right cavernous sinus invasion (Knosp grade II, yellow arrow).

MRI demonstrated a well-defined sellar lesion predominantly occupying the right side of the pituitary fossa with suprasellar and parasellar extension. The lesion extended into the right cavernous sinus (Knosp grade II) and displaced the pituitary stalk toward the left. On MRI, the tumor was iso- to hypointense on T1-weighted images, predominantly isointense on T2-weighted sequences, and demonstrated heterogeneous contrast enhancement following gadolinium administration (Figure 1). No radiological features were considered sufficiently specific to suggest a diagnosis other than an NF PA [6].

## 3. Results

**Figure 2:**
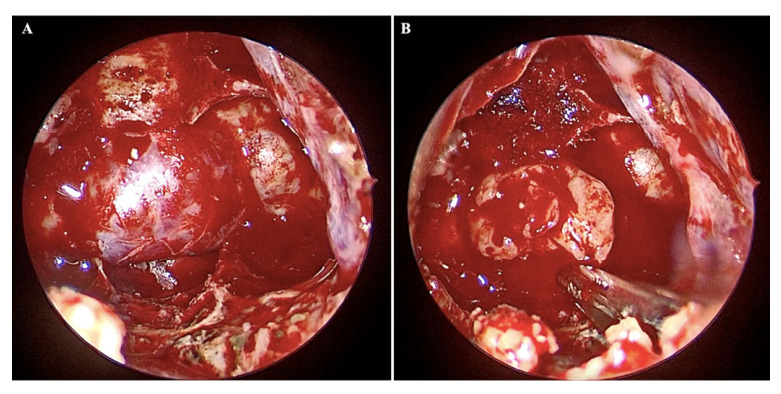
**Intraoperative and macroscopic appearance of the tumor.** (**A**) Endoscopic intraoperative view demonstrating a firm and hypervascular tumor with significant venous bleeding. (**B**) Resected tumor specimen showing irregular, yellow-brown fragments with mixed soft and fibrous consistency.

The patient underwent a binostril endoscopic endonasal transsphenoidal resection. After opening the sellar dura, the lesion demonstrated an atypical intraoperative appearance characterized by marked hypervascularity and firm fibrous consistency. Significant venous bleeding originating predominantly from the medial wall of the right cavernous sinus required meticulous hemostatic control using oxidized regenerated cellulose, gelatin–thrombin matrix, and oxidized cellulose polymer. These intraoperative findings prompted reconsideration of the presumed diagnosis. Despite the vascularity of the lesion, gross total resection (GTR) was achieved based on the intraoperative assessment (Figure 2). No cerebrospinal fluid leakage occurred, and the postoperative course was uneventful. The patient was discharged on postoperative day four.

**Figure 3:**
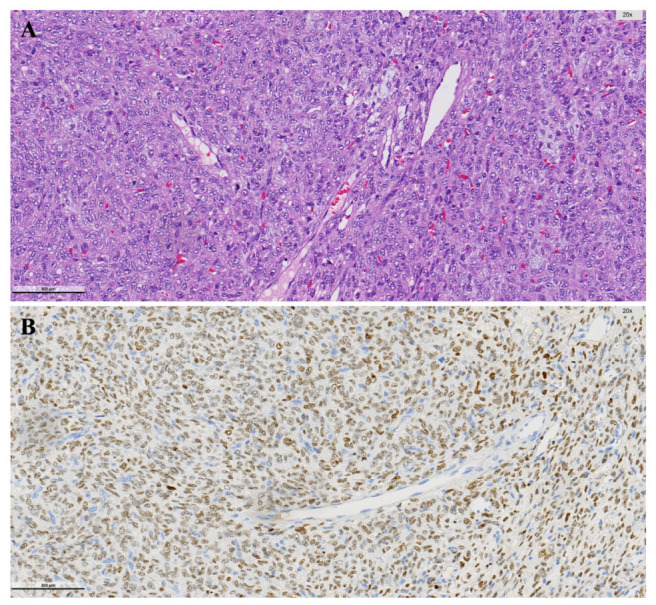
**Histopathological findings of the tumor.** (**A**) Hematoxylin and eosin staining demonstrating a spindle-cell neoplasm with a hemangiopericytoma-like vascular pattern (staghorn vessels) (original magnification ×200). (**B**) Immunohistochemical staining showing strong nuclear STAT6 positivity, confirming the diagnosis of solitary fibrous tumor (DAB stain, ×200 magnification).

Histopathological examination demonstrated a hypercellular spindle-cell neoplasm composed of uniform cells with vesicular nuclei arranged in a patternless architecture and associated with numerous branching, thin-walled (“staghorn”) vessels (Figure 3A). Tumor necrosis was absent. Mitotic activity reached 6 mitoses per 10 high-power fields (HPF), and the Ki-67 labeling index was approximately 15%. Immunohistochemically, the tumor showed diffuse nuclear STAT6 positivity, confirming the diagnosis of solitary fibrous tumor (Figure 3B). Sparse S-100 positivity was observed, while the remaining immunophenotypic findings were consistent with SFT. To exclude other spindle-cell neoplasms of the sellar region, immunohistochemical staining for SSTR2, TTF-1, and synaptophysin was also performed. The absence of immunoreactivity supported exclusion of meningioma, pituicytoma, and neuroendocrine tumors. According to the 2021 WHO Classification of Tumors of the CNS, the lesion was classified as CNS WHO grade 2, based on increased mitotic activity in the absence of tumor necrosis.

Given the absence of residual disease on postoperative MRI, immediate adjuvant radiotherapy (RT) was not recommended after multidisciplinary discussion. Instead, the patient entered a structured surveillance program consisting of serial pituitary MRI and endocrinological follow-up.

**Figure 4:**
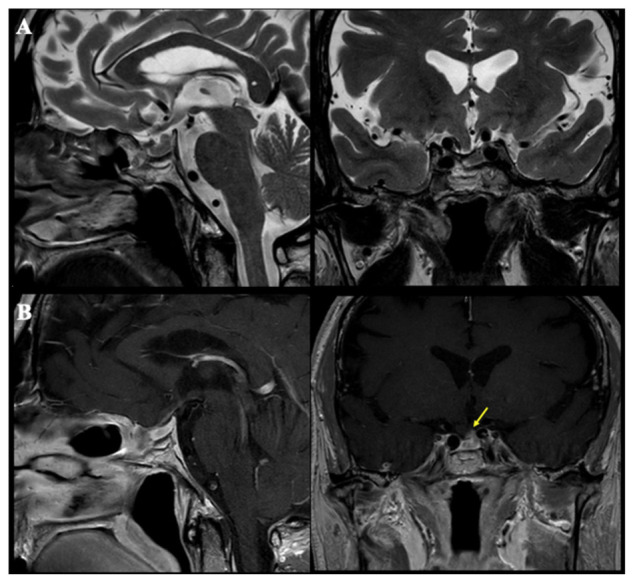
**One-year postoperative magnetic resonance imaging.** (**A**) Sagittal (**left**) and coronal (**right**) T2-weighted images demonstrate the postoperative appearance of the sellar region without evidence of recurrent mass. (**B**) Contrast-enhanced sagittal (**left**) and coronal (**right**) T1-weighted images demonstrate a stable 3 × 2 mm nodular enhancing focus at the operative site (yellow arrow), without interval progression or radiological evidence of tumor recurrence.

At the 12-month follow-up, the patient reported marked clinical improvement, including resolution of headache and visual symptoms. Neurological examination was unremarkable, and follow-up MRI demonstrated no evidence of local recurrence. A small (3 × 2 mm) nodular enhancing focus at the operative site remained unchanged compared with the immediate postoperative examination and was interpreted as stable postoperative change rather than residual or recurrent tumor (Figure 4). Hormonal function remained stable under appropriate replacement therapy [Total testosterone: 122 ng/dL (Range: 193–740); Prolactin: 23.6 ng/mL (Range: 3.46–19.4); Morning 08:00 cortisol: 12.1 µg/dL; Adrenocorticotrophic hormone: 25.5 pg/mL; Thyroid-stimulating hormone: 0.954 µIU/mL; Free thyroxine: 1.07 ng/dL].

## 4. Discussion

SFTs arising in the sellar region represent an exceptionally rare subgroup of intracranial mesenchymal neoplasms [1]. Because only isolated case reports and small case series have been published, their clinicoradiological spectrum remains incompletely characterized, and preoperative diagnosis continues to be challenging [6,7,8]. The structured analysis of previously reported cases summarized in Table 2 demonstrates a remarkably consistent diagnostic pattern characterized by nonspecific clinical presentation, overlapping radiological findings, and definitive diagnosis only after histopathological examination. The present case follows this recurring diagnostic pattern and further illustrates the limitations of conventional preoperative assessment.

One of the most important observations from both the present case and the available literature is the striking similarity between sellar SFTs and NF PAs. Headache, progressive visual impairment, and variable degrees of hypopituitarism constitute the most frequently reported presenting manifestations, reflecting compression of the optic apparatus and normal pituitary gland rather than tumor-specific biological behavior [6,15]. Consequently, clinical findings alone rarely provide meaningful clues for distinguishing SFT from more common sellar lesions [4].

One of the principal reasons for diagnostic uncertainty is the lack of pathognomonic MRI findings. Most reported sellar SFTs demonstrate iso- to hypointense signal intensity on T1-weighted images, variable T2-weighted signal intensity, and heterogeneous contrast enhancement, findings that overlap considerably with PAs, meningiomas, and other sellar tumors [9,10]. The cases reviewed in the present study similarly failed to identify any reproducible MRI feature with sufficient specificity to permit a confident preoperative diagnosis. Nevertheless, certain imaging features—including marked contrast enhancement, flow-voids suggestive of hypervascularity, relatively low T2 signal intensity related to collagen-rich stroma, and reduced diffusion in highly cellular lesions—may collectively increase diagnostic suspicion when interpreted in the appropriate clinical context [11].

Advanced MRI techniques may provide additional diagnostic information, although current evidence remains limited. Susceptibility-weighted imaging may demonstrate prominent intratumoral vessels or susceptibility effects related to hypervascularity, whereas dynamic contrast-enhanced MRI can better characterize tumor perfusion patterns. Diffusion-weighted imaging and apparent diffusion coefficient mapping may reflect tumor cellularity, while magnetic resonance spectroscopy may demonstrate increased choline peaks with reduced N-acetylaspartate [16]. However, none of these techniques has yet been validated as a reliable discriminator between sellar SFTs and PAs. Accordingly, these techniques should be regarded as complementary tools that may increase diagnostic suspicion rather than establish the diagnosis.

Although preoperative imaging is often inconclusive, intraoperative findings may provide valuable diagnostic clues. Hypervascularity and firm fibrous consistency were the most consistently described operative characteristics among cases in which intraoperative observations were reported [17,18]. Similar findings were encountered in the present patient, in whom unexpected cavernous sinus bleeding and dense fibrous tumor consistency prompted reconsideration of the initial diagnosis. While these features are not pathognomonic, their recognition may alert the neurosurgeon to an alternative pathology, facilitate meticulous hemostatic preparation, and encourage close communication with the neuropathologist during intraoperative consultation.

Definitive diagnosis depends on histopathological and immunohistochemical evaluation [14,19]. The identification of diffuse nuclear STAT6 expression, reflecting the underlying NAB2–STAT6 gene fusion, is now regarded as the diagnostic hallmark of SFT and represents the most sensitive and specific immunohistochemical surrogate currently available [2]. Nevertheless, STAT6 should always be interpreted in conjunction with the characteristic morphological features, including spindle-cell proliferation with a patternless architecture and branching (“staghorn”) vasculature. Additional markers such as CD34, BCL-2, and CD99 may provide supportive evidence but lack sufficient specificity to establish the diagnosis independently.

An important issue highlighted by the present revision concerns tumor grading. According to the 2021 WHO Classification of Tumors of the CNS, CNS SFT grading is determined by mitotic activity and tumor necrosis [3]. Tumors demonstrating five or more mitoses per 10 HPFs without necrosis are classified as CNS WHO grade 2, whereas grade 3 requires both elevated mitotic activity and necrosis. Our case fulfilled the criteria for CNS WHO grade 2 because six mitoses per 10 HPFs were identified in the absence of necrosis. This distinction is clinically relevant because WHO grade increasingly informs postoperative surveillance and treatment planning. Although Ki-67 is not incorporated into the formal grading system, increasing proliferative activity has consistently been associated with a higher risk of recurrence and may therefore provide complementary prognostic information [20].

Beyond histological grade, long-term outcome in SFT is influenced by multiple clinicopathological variables rather than any single pathological parameter. The widely adopted risk stratification model proposed by Demicco et al., incorporating patient age, tumor size, mitotic activity, and tumor necrosis, has become an important framework for estimating metastatic risk and guiding postoperative surveillance [21]. Although originally developed for non-meningeal SFTs, the model is increasingly considered applicable to intracranial disease because similar biological determinants appear to underlie recurrence and metastatic behavior.

The optimal postoperative management of sellar SFT remains uncertain because prospective evidence is lacking [1,22]. GTR remains the primary therapeutic objective whenever safely achievable and represents the strongest predictor of local disease control [14,23]. Consistent with larger intracranial SFT/HPC series, adjuvant RT appears to improve local control primarily after subtotal resection or in patients with higher-grade or otherwise high-risk tumors, whereas convincing evidence supporting its routine use following gross total resection of WHO grade 1–2 lesions remains limited [16,24,25]. These studies also demonstrate that delayed local recurrence and extracranial metastasis may occur many years after initial treatment, underscoring the importance of prolonged surveillance regardless of early postoperative imaging findings. Accordingly, current management favors individualized treatment decisions integrating extent of resection, WHO grade, clinicopathological risk factors, anticipated treatment-related morbidity, and the feasibility of long-term follow-up.

In the sellar region, this decision is particularly complex because RT carries unique risks, including optic neuropathy, hypopituitarism, and injury to adjacent neurovascular structures [19,23]. Accordingly, the potential oncological benefit should be carefully balanced against long-term functional morbidity. In the present patient, postoperative MRI confirmed GTR without residual disease. Following multidisciplinary discussion, a surveillance strategy with serial MRI and endocrinological evaluation was favored over immediate postoperative RT. At one-year follow-up, no radiological evidence of recurrence was observed. Although this observation does not establish surveillance as the preferred strategy, it illustrates that postoperative management should be individualized according to extent of resection, pathological risk factors, anticipated treatment morbidity, and the feasibility of long-term follow-up.

An additional strength of the present study is the structured synthesis of all previously reported sellar SFTs. Rather than presenting another isolated case, the diagnostic matrix developed in Table 2 identifies reproducible clinicoradiological and pathological patterns across the available literature. This integrated approach demonstrates that diagnosis depends not on a single imaging characteristic but on the combination of clinical presentation, radiological findings, intraoperative observations, and STAT6-confirmed pathology.

The present study has several limitations. First, this is a single-case report, inherently limiting generalizability. Second, molecular characterization, including identification of the specific NAB2–STAT6 fusion variant and DNA methylation profiling, was not performed. Third, follow-up is currently limited to one year. Nevertheless, integration of the present case with all previously reported sellar SFTs provides the most comprehensive synthesis currently available of the diagnostic challenges associated with this exceptionally rare entity.

Based on the present case together with the cumulative evidence from the published literature, several practical conclusions can be drawn. First, SFT should be included in the differential diagnosis of atypical sellar lesions, particularly when imaging demonstrates unusually intense enhancement or when intraoperative findings reveal a hypervascular, firm tumor inconsistent with a typical PA. Second, definitive diagnosis requires integration of morphology with STAT6 immunohistochemistry in accordance with the contemporary WHO classification. Finally, postoperative management should be individualized according to the extent of resection, pathological risk factors, and feasibility of long-term surveillance rather than the routine application of adjuvant RT.

**Table 2:** Diagnostic and management matrix of previously reported sellar solitary fibrous tumors.

Author	Year	Age/Sex	Symptoms	Endocrine Dysfunction	MRI Findings (T1/T2/Enhancement)	Initial Clinical Diagnosis	Intraoperative Findings	Terminology	STAT6	WHO Grade	Extent of Resection	Adjuvant Therapy	Follow-Up/Recurrence
Cassarino et al. [26]	2003	54/F	Headache, visual disturbance	NR	Solid enhancing intrasellar mass	PA	NR	SFT	NR	NR	GTR	None reported	NR
Pakasa et al. [7]	2005	66/F	Visual disturbance	NR	Solid enhancing intrasellar mass	PA	NR	SFT	NR	NR	GTR	None reported	NR
Kim et al. [27]	2005	56/M	Visual disturbance	NR	NR	PA	NR	SFT	NR	NR	GTR	None reported	NR
Macfarlane et al. [17]	2005	33/M	Headache, visual disturbance	NR	Lobulated parasellar/sellar/suprasellar mass with dural tail	Meningioma/PA	Hypervascular, firm	SFT	NR	NR	STR	None reported	NR
Juco et al. [15]	2007	18/F	Visual disturbance, gait imbalance	No major hormonal excess reported	Sellar mass with suprasellar extension	PA	Dense, rubbery, highly vascular gray semisolid tumor	HPC	NR	NR	STR	None reported	NR
Jalali et al. [28]	2008	35/M	Headache, bilateral visual decline	NR	Suprasellar mass	Craniopharyngioma/PA	NR	HPC	NR	NR	STR	RT	NR
Furlanetto et al. [6]	2009	28/M	Visual disturbance, nocturia	Hypopituitarism/possible partial DI	Heterogeneous T1 and T2/ Strong heterogeneous enhancement.	PA	NR	SFT	NR	NR	GTR	None	No recurrence
Das et al. [29]	2010	47/M	Sudden bilateral visual disturbance, headache	NR	NR	PA	Hypervascular	Malignant HPC	NR	NR	STR	RT	NR
Yin et al. [10]	2010	32/M	Headache, ophthalmalgia, visual disturbance	Hypoglycemia; no pituitary hormone excess	Iso T1/Hyper T2 with focal hypo areas/Homogeneous enhancement	PA	NR	Atypical SFT	NR	2 (historical classification)	GTR	RT	No recurrence
Jain et al. [30]	2012	63/M	Headache, visual disturbance	NR	NR	PA	NR	SFT	NR	NR	GTR	None reported	NR
Wu et al. [31]	2012	53/F	Decreased visual acuity	Mild prolactin elevation	Mixed iso T1/mixed iso-hyper T2/Heterogeneous enhancement	PA	NR	SFT	NR	NR	GTR	None reported	No recurrence
Zhong et al. [11]	2013	25/M	Right visual impairment	No hormonal abnormality	Iso T1/Iso T2/Heterogeneous enhancement	PA	Well-circumscribed, dural-based tumor	SFT	NR	NR	GTR	None	No recurrence
Esquenazi et al. [32]	2014	51/M	Visual disturbance	NR	NR	Sellar mass	Lipomatous, highly vascular lesion	Lipomatous HPC	NR	NR	STR	None reported	NR
Yang et al. [9]	2015	20/F	Headache, visual impairment	Mild hyperprolactinemia	Iso T1/Mixed low-high T2/Heterogeneous enhancement	PA	Firm, highly vascular tumor	SFT	NR	NR	GTR	None	No recurrence
	2015	22/M	Visual impairment	None	Iso T1/Inhomogeneous T2/Homogeneous enhancement	PA	Firm, highly vascular tumor	SFT	NR	NR	GTR	None	No recurrence
Sahai et al. [23]	2016	60/M	Progressive bilateral visual decline	Low T3, low TSH	Iso T1/Iso T2/Intense enhancement	PA	Firm, vascular lesion	SFT	NR	NR	STR	RT	Stable residual
Gibson et al. [33]	2017	34/M	Visual disturbance	NR	NR	PA	NR	Anaplastic HPC	NR	3	GTR	RT	No recurrence
Nesaratnam et al. [34]	2017	73/F	Headache, visual disturbance	NR	NR	PA	NR	SFT/HPC	NR	3	STR	RT	NR
Ghanchi/Patchana et al. [35]	2020	12/M	Headache, progressive visual impairment	Polydipsia without overt DI	Homogeneously enhancing sellar lesion	PA	Firm/fibrous with hypervascular areas	SFT/HPC	Positive	2	GTR	RT	No recurrence
Gunasekaran et al. [18]	2020	69/F	Visual decline, nausea, vomiting	Low ACTH/cortisol	Heterogeneously enhancing cystic sellar–suprasellar mass	PA	Firm, highly vascular	SFT	Positive	2	STR	RT	Stable disease
Thapa et al. [14]	2021	87/F	Headache, visual field defect	Low cortisol, low free T4, mild prolactin elevation	Iso T1/Iso-hyper T2/Heterogeneous enhancement	PA	Markedly vascular, hard, brownish-gray tumor; not aspiratable	SFT/HPC	Positive	2	STR	GKRS	Stable residual
Ma et al. [1]	2023	43/F	Blurred vision	No pituitary hormone abnormality	Iso T1/Iso T2/Enhanced sellar mass; later cavernous sinus invasion	PA	Right portion soft and suctionable; left portion firm and not suctionable	SFT	Positive	2	GTR	Proton therapy (after recurrence)	Local recurrence → controlled after proton therapy
Ebrahimzadeh et al. [36]	2024	54/M	Progressive blurred vision, headache	No hormonal abnormality	Iso T1/Hyper T2/Avid enhancement	PA	Soft grayish mass; peripheral bone invasion/erosion	HPC (SFT/HPC)	Positive	2	GTR	None	No recurrence
Persico et al. [19]	2025	62/F	Incidental sellar mass during encephalopathy workup	Endocrine workup unremarkable	Iso T1/Central T2 hyperintensity/Heterogeneous solid-cystic enhancement	PA	Hypervascular, firm, adherent tumor with brisk bleeding	SFT	Positive	3	GTR	Proton therapy	No recurrence (reported follow-up)
Present case	2026	65/M	Headache, visual impairment, fatigue	Anterior hypopituitarism	Iso–hypo T1/Iso T2/Heterogeneous enhancement	PA	Hypervascular, firm, bleeding	SFT	Positive	3	GTR	Observation (no adjuvant RT)	No recurrence at 12 months

**Notes:** Terminology reflects the original reports. Cases originally reported as hemangiopericytoma (HPC) were interpreted according to the current WHO classification whenever sufficient clinicopathological information was available. Data regarding the extent of resection, adjuvant therapy, and follow-up were extracted from the original publications. When these data were unavailable, the corresponding field was designated as NR (not reported). Follow-up refers to the latest clinical and/or radiological outcome reported by the original authors. **Abbreviations:** GTR, gross total resection; STR, subtotal resection; RT, radiotherapy; GKRS, Gamma Knife radiosurgery; SFT, solitary fibrous tumor; HPC, hemangiopericytoma; PA, pituitary adenoma; NR, not reported.

## 5. Conclusions

Sellar SFTs are exceptionally rare neoplasms that frequently mimic NF PAs, making accurate preoperative diagnosis highly challenging. Because clinical presentation and conventional MRI findings are largely nonspecific, definitive diagnosis requires integration of radiological, intraoperative, histopathological, and immunohistochemical findings, with nuclear STAT6 expression serving as the diagnostic cornerstone.

By integrating the present case with all previously reported sellar SFTs, this study identifies a reproducible diagnostic pattern characterized by nonspecific imaging, intraoperative hypervascularity and fibrous consistency, and definitive postoperative pathological confirmation. Increased awareness of these features may facilitate earlier recognition of this uncommon entity and improve multidisciplinary diagnostic decision-making.

From a therapeutic perspective, GTR should remain the primary surgical objective whenever safely achievable. The present case further suggests that, in carefully selected patients with complete tumor resection and no radiological evidence of residual disease, close radiological and endocrinological surveillance may represent a reasonable alternative to immediate postoperative RT. However, treatment decisions should remain individualized until larger multicenter studies with long-term follow-up establish evidence-based recommendations for the management of sellar SFTs.

## Figures and Tables

**Table 1:** Preoperative endocrine profile demonstrating anterior hypopituitarism.

Test	Results	Range
Testosterone	<0.025 ng/ml	2.84–8.0
Prolactin	0.223 ng/mL	1.4–14.6
Growth hormone (GH)	0.1 ng/mL	0.05–3.00
Insulin-like growth factor-1 (IGF-1)	53.9 ng/mL	40–225
Morning 08:00 cortisol	7.42 µg/dL	6.02–18.4
Adrenocorticotropic hormone (ACTH)	12.49 pg/mL	6–36
Thyroid-stimulating hormone (TSH)	0.246 µIU/mL	0.27–4.2
Free thyroxine (fT4)	0.87 ng/dL	0.93–1.7
Free triiodothyronine (fT3)	1.96 pg/mL	2–4.4

## Data Availability

The original contributions presented in this study are included in the article. Further inquiries can be directed to the corresponding author.

## Abbreviations

CNS	Central nervous system;
GTR	Gross total resection;
HPC	Hemangiopericytoma;
HPF	High-power fields;
MRI	Magnetic resonance imaging;
NF	Non-functioning;
PA	Pituitary adenoma;
SFT	Solitary fibrous tumor;
WHO	World Health Organization;
RT	Radiotherapy.
